# The perceived feasibility of methods to reduce publication bias

**DOI:** 10.1371/journal.pone.0186472

**Published:** 2017-10-24

**Authors:** Harriet A. Carroll, Zoi Toumpakari, Laura Johnson, James A. Betts

**Affiliations:** 1 Department for Health, University of Bath, Claverton Down, Bath, United Kingdom; 2 Centre for Exercise, Nutrition and Health Sciences, School for Policy Studies, University of Bristol, Bristol, United Kingdom; Centre for Research and Technology-Hellas, GREECE

## Abstract

Publication bias is prevalent within the scientific literature. Whilst there are multiple ideas on how to reduce publication bias, only a minority of journals have made substantive changes to address the problem. We aimed to explore the perceived feasibility of strategies to reduce publication bias by gauging opinions of journal editors (n = 73) and other academics/researchers (n = 160) regarding nine methods of publishing and peer-reviewing research: mandatory publication, negative results journals/articles, open reviewing, peer-review training and accreditation, post-publication review, pre-study publication of methodology, published rejection lists, research registration, and two-stage review. Participants completed a questionnaire asking both quantitative (multiple choice or Likert scales) and qualitative (open-ended) questions regarding the barriers to implementing each suggestion, and their strengths and limitations. Participants were asked to rate the nine suggestions, then choose the method they felt was most effective. Mandatory publication was most popularly selected as the ‘most effective’ method of reducing publication bias for editors (25%), and was the third most popular choice for academics/researchers (14%). The most common selection for academics/researchers was two-stage review (26%), but fewer editors prioritised this (11%). Negative results journals/articles were the second and third most common choices for academics/researchers (21%) and editors (16%), respectively. Editors more commonly chose research registration as ‘most effective’ (21%), which was favoured by only 6% of academics/researchers. Whilst mandatory publication was generally favoured by respondents, it is infeasible to trial at a journal level. Where suggestions have already been implemented (e.g. negative results journals/articles, trial registration), efforts should be made to objectively assess their efficacy. Two-stage review should be further trialled as its popularity amongst academics/researchers suggests it may be well received, though editors may be less receptive. Several underlying barriers to change also emerged, including scientific culture, impact factors, and researcher training; these should be further explored to reduce publication bias.

## Introduction

Publication bias is when published research is systematically unrepresentative of all completed studies [1]. The reasons for this are multi-factorial and include influence from industry/funding bodies, editors/reviewers rejecting and/or authors not submitting research on the basis of the results rather than the methodological quality [2,3] in some cases due to the fear of rejection attributed to negative results [4]. Studies have demonstrated the high prevalence of publication bias [5], as well as the potential dangers of having skewed literature (such as the risks outweighing the benefits of a treatment [6]), particularly with regards to clinical practice [3,6].

**Fig 1:**
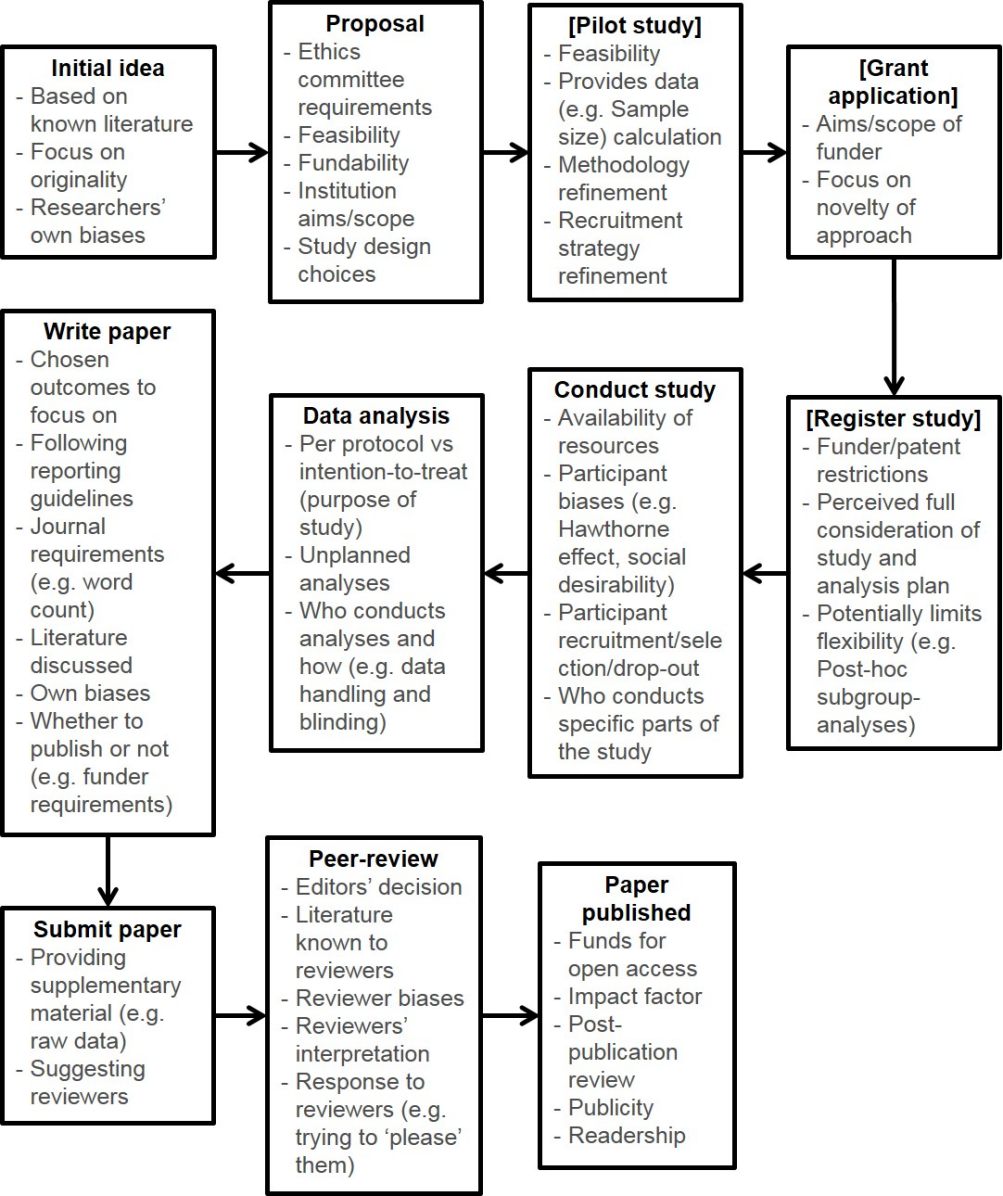
Examples of the potential sources of bias during the research process.

Bias has the potential to occur at several points of the research process (Fig 1) and no one party is solely to blame [3]. Some aspects are difficult to tackle, such as natural human biases [7]. However, other aspects can be addressed with less difficulty, such as the publication and peer-review process [8], which primarily deals with reporting and disseminating research. If biases are mitigated at this stage, then the overall research process may also be improved.

Some organisations and journals have actively tried to reduce publication bias, whilst several studies have aimed to identify barriers towards fixing the problem as well as solutions (e.g. [9–11]). Song et al. [12] discussed several aspects of publication which are related to publication bias, including research sponsor guidelines, trial registration, right to publication, peer-review, conflict of interest disclosure, e-publication and open access. A recent systematic review explored the efficacy of these ideas highlighted by Song et al. [12], finding that they were largely ineffective at reducing publication bias, though the quality of the evidence was low due to difficulties in appropriate study design and implementation [13]. The reasons for this lack of efficacy are likely to be multifactorial owing to the complex nature of publication bias [9].

Whilst studies exploring publication bias provide insight into the current problem and offer potential solutions, to our knowledge none have investigated the perceived feasibility of such solutions by the people that will have to use or implement them. Low perceived feasibility by academics or journal editors may present a barrier to implementing change. In other words, if researchers and/or editors do not want to engage with a new method, it is likely to be ineffective [14].

We therefore aimed to explore different scientific opinions regarding a selection of nine suggested solutions that are currently being used or have been proposed to reduce publication bias, identified from a scoping review of the literature (Table 1). We also investigated the perceived barriers to making such changes. Many previous studies on publication bias and related issues have focused on specific groups, e.g. trialists [15] or editors [11]. We aimed to compare whether there were differences in opinions of editors versus other academics/researchers, as well as whether there were differences between early-career (< 10 y experience) and established (≥ 10 y experience) researchers. These are important comparisons as editors have a crucial role in the dissemination of evidence and make important initial decisions as to whether pass on manuscripts for review [8], established researchers have succeeded in their career via the current publication paradigm [16], and early-career researchers are trying to make a career in the current paradigm.

**Table 1:** Suggestions to reduce publication bias^a^, and examples of where each suggestion is being used and/or who has suggested/advocated the approach.

Suggestion	Definition (as given in the survey)	Studies/authors who have posited this method, or current examples
Mandatory publication	As part of gaining ethical approval and/or by law, researchers would have to guarantee publication of their research, regardless of the findings	Advocated by [17]; [10]
Negative results journals/articles	Having more journals specifically designed to accept research with negative, null and unfavourable results	[11,18,19]
Open reviewing	Requiring that journals name the reviewers and publish their comments with the final manuscript	[10,20]
Peer-review training and accreditation	Requiring all peer-reviewers to attend peer-review training after which they would become accredited peer-reviewers on a peer-review database, which can also highlight potential conflicts of interest	[9,10]
Post-publication review	Editors make a decision regarding the publication of an article. After publication, other researchers provide review comments which the authors can respond to. Although specific experts can be asked to conduct post-publication review, anyone is free to comment on all or part of the paper	[10,16,21]
Pre-study publication of methodology	Researchers publish full details of their planned methodology before commencing the research. The methods are then peer-reviewed to help ensure they are well justified. Once the study is completed, the full manuscript is peer-reviewed and published, regardless of the findings	[10,22]
Published rejection lists	Journals would openly archive the abstracts of rejected manuscripts with a summary of why the paper was rejected	[23]
Research registration	Researchers would be required to register their research on specific databases within a certain time frame of commencing the research. Registration would be compulsory for all research, and would include key aspects of the study design, including the primary and secondary outcomes and analysis plans	Advocated by [17], some ethics committees and required by some journals (e.g.[24])
Two-stage review	Authors initially submit only their introduction and methods to a journal. These get peer-reviewed, after which a decision is made regarding the study quality. If provisionally accepted, the authors would then submit the results and discussion for review. Rejection at this second stage would be justified by concerns over the quality of the reporting/interpreting of the results, but not according to the significance/direction of the results	[8,9,25–27]

^a^The suggestions and examples provided in this table are not extensive, but these ideas were the focus of this study

## Methods

### Sample

In this cross-sectional survey two main groups were recruited, one being journal editors, and the other being academics/researchers. Journal editors included the Editor-in-Chief, Associate and Assistant Editors, and those on Editorial Boards. Editors were recruited via an email to the Editor-in-Chief or similar (e.g. the managing editor) asking them to complete the survey and forward it on to their editorial board. If an editor did not respond to our email, two more reminders were sent, approximately monthly. The journals to contact were chosen via the first 100 articles to come up in a Google Scholar, Jane Biosemantics and PubMed search of ‘publication bias’. Additionally, the top 100 journals according to impact factor (assessed using http://www.scimagojr.com/journalrank.php rankings relating to 2014) were contacted. All searches were conducted in July 2015. The editors (n = 157) of 111 journals were contacted. Of these, editors from three journals replied declining to participate. An additional 10 editors from journals that were not directly contacted completed the survey. Impact factors of the journals contacted ranged from 0.12 to 45.22 (median 2.80, interquartile range [IQR] 1.41, 7.97).

‘Academics/researchers’ included those who self-identified as being involved in research (past or present), including those with peer-reviewed published research, postgraduate students, lecturers/professors (with and without research) and full-time researchers. Although the sampling strategy was mainly focused in academia, those working in industry were not excluded.

For ‘academics/researchers’, convenience snowball sampling was used. Dissemination was conducted via email and Twitter based on the authors’ own networks. In order to help broaden the scope of the research, some prominent scientists (those who have published papers about publication bias and/or flaws in the research process, n = 37) were also contacted directly to complete and help disseminate the surveys. To gauge representativeness, global university rankings were used (https://www.timeshighereducation.com/world-university-rankings/2015/world-ranking#!/page/0/length/25) to rank respondents from the top 500 universities (those not in the top 500 were ranked ‘500’). All respondents in both surveys were required to give online written informed consent before the survey would allow them to continue.

### Study design

Two online surveys were created using Bristol Online Surveys (University of Bristol, Bristol, UK): one for editors and one for academics/researchers. The two surveys were virtually identical, with only two key differences. Firstly, some questions were modified in order to suit the respondent, for example “I would consider this for my journal” was replaced with “If a journal implemented this, I would be more inclined to submit to them”, accordingly. Secondly, editors were asked to provide the name of their respective journal, whereas other academics/researchers were required to provide their current/most recent institution. Further demographic questions were added to the survey for other academics/researchers, such as stating their current job role.

The surveys were created by three of the study authors (HAC, LJ, JAB) specifically for this research. Whilst the surveys did not undergo any formal validation, the questions chosen were based off previous research investigating similar topics with additional questions added as appropriate in order to tailor the survey to fit our research questions. The nine proposed solutions were based on recent innovations by journals or suggestions made by researchers in the literature (highlighted in Table 1). The initial questions regarding characteristics of participants (e.g. institutional affiliation) were based off the online questionnaire disseminated by Scherer and Trelle [28], and Malicki et al. [10]. Although Malicki et al. gave qualitative options as they were investigating primarily one proposal to reduce publication bias, we opted for Likert scales in order to reduce the time-burden on respondents due to enquiring about several proposals. We then allowed adequate space for open ended responses, more in line with the style of questions asked by Kien et al. [9] who used semi-structured interviews asking key stakeholders questions regarding publication bias.

The surveys provided suggestions (Table 1) on how to prevent publication bias, which respondents had to rate using a Likert scale (where 1 = not at all effective and 5 = extremely effective). Participants were then asked to choose the method they felt would be most effective, and consider specific barriers that may prevent it from being implemented. Respondents were also given opportunities to comment on any of the other suggestions, as well as provide their own ideas. In order to try and keep the survey as short as possible, all open ended responses were optional, as were some non-core quantitative questions (e.g. “What factors influence your choice of journal for publication?”). Copies of the survey questions are provided in the Supplementary Material S1 and S2 Files.

### Analysis

Quantitative data were visually checked for normality using a histogram, and either means (± standard deviation [*SD*]) or medians (IQR) are reported as appropriate. Chi squared, Kruskal-Wallis or independent samples *t*-test were used as necessary to test whether there were significant differences in opinions between editors and academics/researchers, or significant differences between early career academics/researchers and established academics/researchers. Data were analysed using SPSS (version 22, IBM, Armonk, New York, USA).

Open-ended answers were analysed using thematic analysis [29]. Similar answers were grouped to create themes for each question. Prevalent themes across the survey were also coded to create broader overall themes. Two researchers (HAC and ZT) independently analysed the data, and differences were discussed in order to come to an agreement when there were discrepancies in the coding. Coding was done manually (no specialist software was used). This study was ethically reviewed and approved by the Research Ethics Approval Committee for Health at the University of Bath (ref: EP 14/15 216).

## Results and discussion

Seventy-three editors responded to our survey. Impact factors for their respective journals ranged from 0.14 to 45.22 (median 2.06, IQR 1.10, 3.02), showing more lower-impact journals responded. A further 160 academics/researchers also responded. Characteristics of the academics/researchers who responded are presented in Table 2. Respondents were from a wide range of institutions, ranking from within the top ten globally (4%) to outside the top 500 (26%) (median 201, IQR 74, 500). Most respondents were from the UK (n = 102 [64%] academics/researchers) reducing the global representativeness of the opinions obtained presenting a limitation of the convenience sampling strategy used. Whilst academics from several other countries also completed the survey, such as Australia (n = 5), Israel (n = 1), the Netherlands (n = 9) and the US (n = 10), no respondents were from countries within Africa or Asia. Future work would therefore need to corroborate our findings (from predominantly UK and European researchers) to ensure they represent opinions of international and underrepresented researchers, particularly in continents with growing numbers of academics such as Africa and Asia.

**Table 2:** Characteristics of academics/researchers (n = 160).

	Total (n = 160)	Experience < 10 y (n = 98)	Experience ≥ 10 y (n = 62)
Median global institutional ranking (IQR)	201 (74, 500)	201 (74, 314)	289 (74, 500)
Highest qualification PhD or MD (%)	73	60	92
Lecturers/Professors^a^ (%)	29	14	52
MDs	1	1	2
Researchers^b^ (%)	34	32	37
PhD students (%)	28	44	3
Masters’ students (%)	3	4	0
Other (%)	6	5	7
Conducted systematic-review/meta-analysis (%)	49	37	68
Mainly quantitative (%)	60	61	58
Mainly qualitative (%)	9	11	5
Mixed methods (%)	31	28	37
Medicine and related subjects (%)	50	40	66
Social sciences and law (%)	30	37	19
Science and mathematics (%)	16	17	13
Engineering and technology (%)	4	5	2
Other (%)	1	1	0
Had been funded by industry (%)	44	33	61
Published in peer-reviewed journal (%)	84	75	98
Median (IQR) impact factor of journals respondents have published in	1.52 (1.17, 2.16)	1.42 (1.17, 2.12)	1.62 (1.19, 1.88)
Conducted peer-review (%)	67	52	90
Median (IQR) impact factor of journals respondents have peer-reviewed for	1.49 (1.21, 1.94)	1.44 (1.21, 2.18)	1.56 (1.23, 2.10)

Abbreviations: IQR, interquartile range; MD, Doctor of Medicine

^a^Lecturers includes both teaching only and teaching with research

^b^Researchers includes: post-doctoral researchers, research assistants/associates/fellows and full-time researchers

Both editors and academics/researchers were largely in agreement as to why people choose to publish in (their) journals (Table 3). Journal quality was ranked similarly highly by both editors and academics/researchers (*P* = 0.230), but most appropriate content was ranked slightly higher by academics/researchers than editors (*P* = 0.031).

**Table 3:** Median ranking of reasons as to why people choose to publish in a journal.

			Academics/researchers^b^							
Reason	Editors^a^	%^c^	All academics/researchers	%^c^	*P* editors vs academics^d^	Experience < 10 y	%^c^	Experience ≥ 10 y	%^c^	*P* experience < 10 y vs ≥ 10 y^d^
Most appropriate content	2	97	1	99	0.031	1	99	1	98	0.992
Journal quality	2	96	2	98	0.230	2	97	2	98	0.282
Open access	4	74	4	88	0.696	3	91	4	51	0.527
Other^e^	3	30	5	25	0.940	5	21	4	29	0.004
Turnaround time	3	8	3	90	0.065	4	90	3	89	0.129

^a^Reasons the Editors believed people chose their respective journal to publish in (median rank)

^b^Reasons respondents chose journals to publish their work in (median rank)

^c^Optional question; % represents the percentage of respondents who provided an answer out of a total sample of n = 73 for editors and n = 160 for total academics/researchers (n = 98 with < 10 y experience and n = 62 with ≥ 10 y experience)

^d^Differences in median rank tested using Kruskal-Wallis

^e^Open ended responses for ‘other’ included: Cost, likelihood of acceptance, audience, independent publishers, quality of review, PubMed indexed, fair editors/reviewers, publication delay and online early access

Most respondents had heard of publication bias (97% of editors and 91% of academics/researchers), though this was lower for early career researchers compared to experienced academics/researchers (87% compared to 98%, respectively; *P* = 0.011; Table 4). Most respondents (89% of editors and 90% of academics/researchers; *P* = 0.823) felt that publication bias was a problem in the literature. A small number of editors (n = 8, 11%) felt publication bias was not a problem with qualitative analysis of open-ended responses suggesting some editors did not believe publication bias to be widespread:

“*I don’t accept that publication bias exists in a widespread fashion as this series of questions suggests*” (Ed44)

**Table 4:** Responses to questions regarding awareness of publication bias and efficacy of peer-review.

	Editors	Academics/researchers				
Question	Total (n = 73)	Total (n = 160)	*P* editors vs academics^a^	Experience < 10 y (n = 98)	Experience ≥ 10 y (n = 62)	*P* experience < 10 y vs ≥ 10 y^a^
Had heard of publication bias (%)	97	91	0.092	87	98	0.011
Felt there is a problem of publication bias in the literature (%)	89	90	0.823	87	95	0.083
Felt peer-review is an effective means of publishing *quality* research (%)	90	79	0.030	80	77	0.743
Felt peer-review is an effective means of publishing *unbiased* research (%)	58	36	0.002	39	32	0.403
Thinks the current system of publication should change to reduce publication bias (%)	75	89	0.009	90	87	0.599

^a^Chi-square test

Whilst most respondents (90% of editors and 79% of academics/researchers; *P* = 0.030) felt the peer-review method of publication is an effective means of publishing *quality* research, far fewer (58% of editors and 36% of academics/researchers; *P* = 0.002) felt it was effective at publishing *unbiased* research (Table 4). These differences between academics/researchers and editors may highlight a more favourable bias towards a publication system in which editors actively contribute to and influence. There were no differences between academics/researchers with < 10 y compared to ≥ 10 y experience for either statement (*P* = 0.743 and *P* = 0.403, respectively).

### Effectiveness of suggestions to reduce publication bias

Whilst each suggestion to reduce publication bias has its own merit, some were deemed more effective at reducing publication bias. Both academics/researchers and editors popularly selected mandatory publication and negative results journals/articles as “most effective” at reducing publication bias (Table 5). A key difference between editors and academics/researchers was that research registration was the second most popular choice for editors, whereas two-stage review was the most popular choice for academics/researchers (Table 5). Similar results were found between academics/researchers with < 10 y and ≥ 10 y experience, though those with > 10 y experience more commonly selected mandatory publication whereas those with < 10 y experience more popularly chose open reviewing as the most effective proposal for reducing publication bias.

**Table 5:** Average scores of how effective respondents think each suggestion will be (Likert scale where 1 = not at all effective and 5 = extremely effective) and number of respondents who selected each suggestion as “most effective” at reducing publication bias.

	Editors (n = 73)		Academics (n = 160)			Academics < 10 y experience (n = 98)		Academics > 10 y experience (n = 62)		
Suggestion	Mean ± *SD* score^a^	Chosen as most effective (%)	Mean ± *SD* score^a^	Chosen as most effective (%)	*p*_diff_ mean scores^b^	Mean ± *SD* score^a^	Chosen as most effective (%)	Mean ± *SD* score^a^	Chosen as most effective (%)	*p*_diff_ mean scores^b^
Research registration	3.3 ± 1.5	21	2.9 ± 1.2	6	0.064	2.9 ± 1.2	6	2.9 ± 1.3	7	0.831
Mandatory publication	3.1 ± 1.6	25	3.0 ± 1.4	14	0.564	3.0 ± 1.4	11	2.9 ± 1.4	18	0.420
Negative results journals/articles	3.1 ± 1.3	16	3.6 ± 1.3	21	0.002	3.9 ± 1.2	24	3.2 ± 1.4	18	0.003
Pre-study publication of methodology	3.0 ± 1.4	8	3.1 ± 1.3	6	0.606	3.2 ± 1.3	7	2.9 ± 1.3	5	0.197
Two-stage review	2.7 ± 1.4	11	3.4 ± 1.3	26	0.001	3.5 ± 1.3	28	3.1 ± 1.4	23	0.070
Peer-review training and accreditation	2.6 ± 1.1	11	3.3 ± 1.3	10	< 0.001	3.4 ± 1.3	11	3.0 ± 1.2	8	0.048
Post-publication review	2.5 ± 1.2	6	2.7 ± 1.2	3	0.201	2.8 ± 1.2	1	2.7 ± 1.2	5	0.521
Published rejection lists	2.2 ± 1.1	1	3.0 ± 1.2	4	< 0.001	3.0 ± 1.2	0	2.9 ± 1.3	10	0.630
Open reviewing	1.9 ± 1.0	1	2.9 ± 1.2	11	< 0.001	3.0 ± 1.2	12	2.9 ± 1.3	8	0.844

Abbreviations: *SD*, standard deviation

^a^Mean scores provided by the whole sample, not just those who selected the suggestion as the most effective

^b^Independent samples t-test

Participants were also asked to rate the effectiveness of each suggestion provided (1–5 Likert scale; Table 5). Both editors and academics/researchers gave a score in the top three for negative results journals/articles, though academics/researchers rated it more highly than editors (*p* = 0.002). The difference in scores for negative results journals/articles between editors and academics/researchers is driven predominantly by the higher score (*p* = 0.003) given by academics/researchers with < 10 y experience.

Nonetheless, these findings may imply that negative results journals/articles are both favoured by respondents and deemed to be comparatively effective. Thus more negative results journals/articles may be well received, with respondents highlighting the importance of negative/null results being published and that this would be an easy suggestion to implement (S1B Table). There were some concerns about implementing this approach more widely though, such as impact factors, willingness of publishers, and whether negative results journals/articles draw more attention to results rather than methodological quality. Many respondents noted that a common perception is that null/negative/unfavourable findings are somewhat different to positive/favourable results and the problems with changing this culture:

“[negative results journals] *somewhat puts research with negative results in a category apart*, *suggesting they are not suitable for the subject-specific journals in that area*” (Ed54)

“*Accepting negative or null results would require a re-definition of originality and scientific contribution to knowledge*” (Ac107)

Future research should therefore qualitatively investigate whether negative results journals/articles do alter perceptions regarding the relative importance of the methodology and results, and quantitatively assess whether this affects publication.

Mandatory publication also scored in the top three for editors (3.1 ± 1.6; Table 5). Whilst academics/researchers scored mandatory publication similarly to editors (3.0 ± 1.4; *p* = 0.564) this score was only the fifth highest and was similar between more and less experienced academics/researchers (*p* = 0.420). Mandatory publication having a comparatively lower score than other suggestions for academics/researchers is possibly due to concerns regarding implementation and enforcement (S1A Table), despite it being popularly selected as “most effective” (Table 5). This is in accordance with (the albeit low quality and limited) evidence that whilst mandatory publication as per the Food and Drug Administration Amendment Act 2007 was effective at increasing the number of results made available, it was ineffective at increasing reported results to an acceptable level (22% of registered trials falling under the Act reported results, compared to 10% of trials which did not fall under the Act) [30], demonstrating the difficulties in enforcement.

There was some concern that implementing a system which enforces publication may negatively impact industry support for research (further supported in S1A Table):

“*If commercial interests commission research then… Forcing publication may result in withdrawal of support of research*” (Ed5)

It could however also be argued that this would also eliminate industry funding containing limits on publication. Equally, some respondents noted that “*Research* [can] *remain unpublished for a variety of legitimate (i*.*e*. *non biasing) reasons*” (Ac47).

Two-stage review was rated second highest for academics/researchers but fifth for editors (3.4 ± 1.3 compared to 2.7 ± 1.4 for editors; *p* = 0.001), supporting the popularity of academics/researcher choosing it as “most effective” at reducing bias (Table 5). This method has been suggested or discussed previously in the literature [9,25–27] with the rationale that reviewers and editors will not be influenced by favourable or positive results, echoed in our findings:

“*This places an emphasis on the quality of research regardless of the direction of the results”* (Ac139)

However, others have noted that this still carries issues, for example the introduction and methods would still be written and submitted after the results are known by the authors, introducing hindsight bias [3]. One academic/researcher noted that a new type of bias may even be created:

“*…statistically significant results have 'news value' and so each decision to publish would become a gamble*. *There may be a resultant bias towards only accepting 'safe bets' and steering clear of 'long shots'…*” (Ac10)

Whilst some also noted that this method may increase time to review and/or workload, *“Two-stage review would create even more delays to an already lengthy process”* (Ac122), others illustrated that reviewer time commitments and workloads could be reduced particularly when it came to rejecting papers (S1I Table):

*“A two-stage review makes it quicker to reject papers that have clear faults*, *because reviewers would only have to read half of the manuscript*.*”* (Ac18)

A previous small pilot study that aimed to test a two-stage review process for both reviewers and editors [8] found that 86% of reviewers would be willing to complete the full review after being given the initial abbreviated paper (introduction and methods only). No details were provided regarding the quality of the reviews or the rejection rate, leaving the effectiveness of this method to reduce publication bias currently untested. Editors were consistent in their assessment of the manuscripts over both stages of the review process 77% of the time; i.e. decisions were generally not altered upon reading the results. In saying this, 7% of positive results articles that were rejected by editors in the first stage of review were accepted during the second stage, which did not occur with negative results articles. Comparatively, one negative results article was initially queued for peer-review but subsequently rejected by the editor upon reading the results. These findings suggest that editors should remain blinded to results when making editorial decisions. Further research should therefore investigate whether reviewers provide less results-biased reviews under a two-stage review system; thus we commend the *BMC Psychology* who have recently announced a pilot study [31], though it does not appear that editors will be results-blinded upon submission despite the aforementioned research suggesting results-blinding editors is important [8]. Testing this method will help determine whether the concerns of our respondents are valid regarding time, workload and different types of bias.

Peer-review training and accreditation was rated third highest by academics/researchers but was less favoured by editors (3.3 ± 1.3 compared to 2.6 ± 1.1 for editors; *p* < 0.001; Table 5). Open ended responses suggested it was viewed positively (e.g. increases review quality) despite the barriers (e.g. extra work for a voluntary role; S1D Table). The differences in scores between editors and academics/researchers is driven by the higher score given by academics/researchers with < 10 y experience (4.3 ± 1.3 compared to 3.0 ± 1.2 for those with > 10 y experience; *p* = 0.048). Taken together, these findings are in accordance with Kien et al. [9] which may suggest that (particularly less experienced) peer-reviewers would be more inclined to engage in training as they believe it will be effective at reducing biases. Despite this, research has shown that peer-review training in itself shows no effect on review quality; studies that do show effects have methodological issues such that the validity and generalisability of the research was compromised [32,33]. No existing study of peer-review training and/or accreditation has specifically focused on reducing publication bias.

Although research registration was rated highly and was the second most popular choice for editors, academics/researchers gave it one of the lowest mean scores for effectiveness (2.9 ± 1.2 compared to 3.3 ± 1.5 for editors; *p* = 0.064) (Table 5), with similar scores between academics/researchers with < 10 y and > 10 y experience (*p* = 0.831). The efficacy of research registration has recently been questioned with evidence showing non-reporting and selective publication of outcomes is still common [4]. Reasons for this are multifactorial; our respondents stated that barriers include resistance to registering trials, increased time and effort, lack of incentive to register a trial and agreeing on standardised registration procedures (S1H Table), with similar opinions expressed in previous research [9].

There was general agreement as to what methods would be least effective. Both editors and academics/researchers felt post-publication review and open reviewing would be ineffective at reducing publication bias (Table 5), with open ended answers showing that these were not perceived to address bias (S1C and S1E Table). Although previous research has found post-publication review to be regarded positively by academics/researchers (e.g. [10,21]), others have highlighted the lack of evidence as to whether it actually reduces bias [14].

There is evidence to suggest that open (or at least unblinded) reviewing is not effective at improving the inter-rater reliability of reviews (notwithstanding the limitations of this metric [34]). Research is mixed regarding the efficacy of open reviewing in terms of review quality; some evidence suggests open reviewing provides no improvement [35–37] whereas others have found some improvement [38] to the quality of reviews. Further, some research suggests open reviewing increases the likelihood of a recommendation to accept the manuscript [38] which may help to reduce publication bias. Nevertheless, the likelihood of reviewers refusing to review a manuscript increases, as does the time to review [35,38], thus it may be worth focusing attention on methods which are perceived as more effective and do not increase the likelihood of reviewers declining to review.

Published rejection lists scored the lowest for editors, supported by only one editor selecting it as the “most effective”. Though it was scored higher by academics/researchers (3.0 ± 1.2 versus 2.2 ± 1.1 for editors; *p* < 0.001), only six (4%) chose it as the “most effective” suggestion at reducing publication bias (Table 5). Whilst some highlighted that this would be one way to get results into the literature, others stated that it may shame authors or reduce the incentive to try to publish research (S1G Table). No research to our knowledge has tested this method; considering its low perceived effectiveness it may be unlikely that researchers will fully engage with the proposal regardless of whether it does reduce publication bias.

### “A new system is needed” (Ac53)

Whilst the majority of both editors and academics/researchers agreed that the current system of publication should change to reduce publication bias, fewer editors (75%) compared to academics/researchers (89%) held the sentiment (*P* = 0.009), with no differences found between early-career and established academics/researchers (*P* = 0.599; Table 4). These results may suggest bias from those who maintain the current system, and/or naivety from academics/researchers who do not fully understand it—particularly the early-career researchers in our sample of whom only 75% had published in a peer-reviewed journal and only 52% had undertaken peer-review (Table 2).

Reasons for being in favour of change included that the current system for publication is not in line with scientific principles, knowledge should benefit the public and not publications or headlines, and that publication bias exists and is problematic (S2 Table).

A key reason why respondents did not support the need for change was the perception that peer-review is the best system available, or that there are no better alternatives:

“*I can’t see any alternative to peer-review” (Ed10)*

*“No one has demonstrated the other systems work better*” (Ac120)

Whilst these are valid points, the current mechanism of peer-review did not develop from theory to testing with adequate comparators [39] as per the usual scientific method. Currently, however, it is often seen as the most acceptable option for research assessment despite the lack of consistent definition, rigorous testing or systematic critique (as highlighted by Smith [40]). This would not hold value in any other aspect of scientific practice:

“*If you were starting from scratch now you wouldn’t design it this way*” (Ac74)

Further, the few studies that have been conducted show little evidence that peer-review consistently finds errors [40] but does provide evidence that the rate of error detection can be skewed by seemingly irrelevant things such as the direction of the findings [41]. Trialling new methods in order to reduce bias and increase fairness within the current peer-review and publication model is therefore important, particularly as our data suggest there is concern that the current publication process is not perceived as effective at publishing unbiased research.

A consistently mentioned issue was that it is a lot of effort to change the system for only a minority of poor or problematic papers (S2 Table):

*“Too difficult and will require everyone to be bothered to fix the problem of a few papers that should be published but won’t because of negative results”* (Ed35)

Whilst we acknowledge that the prevalence and importance of publication bias may differ between disciplines, it is still important to address the issue, particularly in fields such as health and medicine (e.g. Whittington *et al*. [6]). For example, as one academic stated:

“*Mandatory publication alone would have precluded e*.*g*. *the Tamiflu case*” (Ac69) [N.B. Tamiflu is an influenza drug which was thought to be effective at reducing complications arising from influenza infection until withheld trial data was released after several years of requests from researchers to the pharmaceutical company, Roche. Roche generated $18 billion in sales of Tamiflu from healthcare providers believing it to be effective [42]. After the data were released, a Cochrane review found Tamiflu to be ineffective at reducing influenza complications [43]]

Others were more positive making the point that if there are ways to improve science, they should be tested and implemented as appropriate (S2 Table):

“*All attempts to reduce publication bias should be made regardless of the state of the current system*, *it should be an evolving process*” (Ed71)

Furthermore, reducing publication bias can help the overall scientific process; for example, if more failed experiments are reported, other researchers will not spend time and resources making the same mistakes (S1A Table).

### Wider issue: Scientific culture

Whilst the initial purpose of this survey was intended to gauge opinions of different methods of peer-review and publication in order to reduce publication bias, other related issues emerged. These were primarily the scientific culture, impact factors, and researcher training (discussed below). The purpose of presenting these points is to encourage further research into these areas in terms of how they impact bias and identify options for change, particularly as similar themes have emerged in previous research (e.g. [9,10]).

Many respondents commented on the overall culture of science, with a common theme being that the academic expectations and the perceptions of quality science need changing:

“*Scientists should be employed and promoted on the basis of the quality and integrity of their research*, *not by counting the number of publications in prestigious journals and the number of citations*” (Ed61)

“*Crooked academic targets and metrics need to go*” (Ac42)

A particular issue consistently raised regarding scientific culture was regarding (young) academics on short term contracts. Short term contracts can lead to bias as researchers will aim to submit only the most ‘publishable’ results for peer-review at high impact factor journals in order to be competitive for future work:

“*There is too much pressure on academics to publish… If this pressure was relieved*, *researchers would have more opportunities to conduct better research and publish better papers*. *Journals would have more space to publish good research*, *high quality studies with null results*, *replications*, *research plans*, *and so forth*” (Ed56)

Several respondents suggested that academia in itself may not be conducive to change:

*“One major barrier is the resistance to changes in general…”* (Ac38)

*“…researchers have so much going on*, *and so little time*, *it would be very hard to gain support for much change that requires substantial behaviour change/extra tasks”* (Ac96)

Thus trialling the effectiveness of the more favoured suggestions posited in this (and other) research may increase engagement, particularly as two of the most popular ideas (negative results journals/articles and two-stage review) would likely require minimal to no extra work for the majority of academics/researchers.

### Wider issue: Journals and impact factors

The publication process was viewed as flawed and going against scientific principles by many respondents (S2 Table). More specifically, impact factors were frequently mentioned as problematic throughout the surveys:

“*Too much emphasis on impact factors*, *which are in fact fairly meaningless*” (Ac111)

A key theme was that impact factors are partially driven by the need for research to be interesting to the readers:

“*…part of the reason journals exist is that readers are interested in content… I don’t think most people would be too interested in reading a journal of methodology*” (Ed49)

Whilst not a consensus, one academic/researcher provided an alternative perspective that top journals filtering for exciting results was not a problem in itself, but did lead to other problems:

“*As a reader I'm much more interested to learn about the one-in-a-billion discovery of a flying pig in Nature*, *than about the gazzilion mundane swine observations across many labs that were necessary in order for someone to stumble across such a nugget*. *In my view the problem is not so much with (some) journals filtering this way*, *it is with hiring and funding committees over-emphasizing Nature papers in their decisions*. *This creates an incentive to have a very lenient threshold for crying 'flying pig' and to be less critical of our own findings*” (Ac60)

### Wider issues: Training

Whilst peer-review training and accreditation was a fairly well-rated suggestion, it was not perceived to be as effective as other suggestions, which is supported by (limited) research showing minimal to no effect from peer-review training (e.g. [33]). There was, however, a general agreement that training students more in the scientific process would be beneficial:

“*Training at postgraduate level on the implications of publication bias to prevent students adopting bad practice*” (Ac84)

These sentiments have also been highlighted in previous research [9,10] with suggestions such as teaching the ethical responsibility to share data in PhD programmes and addressing publication bias in the curriculum [9]. This consensus may have merit compared to peer-review training as a more holistic approach may be efficacious across multiple facets of the research process compared to short training interventions.

Respondents highlighted several elements of the scientific process which appear to be insufficiently taught formally to students, such as how to write and submit a paper (including reporting guidelines) and peer-reviewing. Teaching such issues to students also addresses some respondents concerns with peer-review training, namely that it would demand additional time from reviewers “*who offer a free service and thus should not be antagonised*” (Ed45). Improved researcher training also has the potential to help prevent other core issues which contribute to bias, such as poor study design:

“*…I have not seen any evidence that high quality research is not being published*. *We are however overrun by poor quality research diluting good science*” (Ed13)

### Causes of and ways to reduce bias

Some respondents offered other ideas to help reduce bias (S3 Table), whilst others highlighted who or what they believe is the cause of publication bias. These wide range of opinions are in accordance with previous research [11] and emphasise the complexity of publication bias, whilst demonstrating that there will not likely be one clear solution. It may therefore be appropriate for several parties to make small changes in order to reduce publication bias and related issues, such as:

- lecturers/professors to teach about publication bias, peer-reviewing and related matters
- researchers to design and conduct high quality research that is reported and published accurately and transparently
- institutions to judge scientists on their research quality (rather than e.g. publishing in high-impact journals) and to offer longer term contracts that would accommodate the lower frequency of publications that higher-quality work typically demands
- editors to send high-quality studies on for review regardless of the findings
- reviewers to focus on methodological and reporting quality
- all involved in science (including publishers and the media) to value and prioritise high-quality evidence

There were some concerns that changing publishing practices does not necessarily impact whether or not research is published—a concern which has also been highlighted by previous research (e.g. [3]):

“*…there are things peer review cannot help*, *for example if unfavourable data was removed*” (Ed71)

Publishing practices are one important aspect of the research process that can hinder publication. As negative results are less likely to get published, and generally take longer to get published [5], researchers may be less inclined to submit these findings, or may be inclined to present favourable results more prominently. This has led to issues such as P-value hacking [44] and hypothesising after the results are known (HARKing) [45]. Thus improving publishing practices may encourage more open reporting and higher willingness to submit research. However we stress the importance of testing any new methods thoroughly to ensure their efficacy. This would likely work most effectively in tandem with changes to the wider issues (scientific culture, journals/impact factors and researcher training) as described above.

Owing to the potential introduction of unintended consequences (e.g. new biases), journals should aim to trial the more feasible options, both in isolation and in combination with other ideas where appropriate. As many noted that some methods would not work well in specific disciplines, it should be the decision of editors to find what ideas would work in their field. Outcomes of trials of publishing practices would need to include predefined markers of study quality (e.g. the Cochrane risk of bias tool); submission and acceptance rates of studies with favourable and unfavourable results; and a measure of the quality of reviewer comments. This could be conducted by randomly assigning submissions to the usual system or a test system and comparing differences; this may however suffer contamination effects if the same editor has to implement multiple systems with different submission processes within the same journal. Alternatively, a blanket introduction of the new system to the journal and retrospectively comparing the pre-defined outcomes.

In the context of this research, we propose that journals further trial two-stage reviewing as this was favoured by academics/researchers (thus is likely to be better received) and has the advantage of being relatively easy to test compared to other favoured suggestions (e.g. mandatory publication). In accordance with previous work, it is important that editors are blinded to the findings too which may create further resistance particularly as editors did not rate two-stage reviewing as highly as academics/researchers. Enforcing any new system was a key concern in both our and others’ research, thus testing methods which are both popular and perceived to be effective may improve self-regulation. Journals that are using other methods (e.g. requiring research registration or negative results journals/articles) should aim to objectively assess the efficacy of these popular and well-rated methods in reducing publication bias.

Similar research is still needed to confirm our findings are representative of both academics/researchers and journal editors, particularly those outside of the UK. Further, our work is limited by the choice of potential solutions proposed; other ideas may have been favoured more than the ones provided. The surveys used were designed to be relatively quick to complete in order to encourage participation. Whilst this led to a relatively high response rate compared to similar research in the field, our findings are comparatively superficial; future work should investigate opinions regarding these issues in more depth, such as qualitative interviews or having a narrower focus. Lastly, whilst understanding opinions is important before wider change, once more extensive research has been conducted on methods to reduce publication bias, perceptions of each method may alter; for example if two-stage reviewing is shown to be successful, editors may view it more favourably, and alternatively if it is found to increase reviewing or publication time, academics/researchers may view it less favourably.

## Conclusion

This survey explored nine suggestions proposed to reduce publication bias. There was some agreement between editors and academics/researchers that certain suggestions may be more effective than others, however not all would be feasible to trial in a study (e.g. mandatory publication). Negative results articles were considered favourably overall, whilst editors preferred research registration and academics/researchers preferred two-stage review. As negative results journals/articles and research registration are already available/required in some journals, efforts should be made to objectively assess the efficacy of these methods in reducing publication bias. Two-stage review should be further trialled by journals to formally assess its efficacy at reducing publication bias as its popularity amongst academics suggests it may be well received.

Other issues surrounding academia and research which relate to publication bias were also highlighted. These included the overall scientific culture, journals and impact factors, and researcher training. Further research should explore these issues with a view to finding feasible solutions to help reduce bias and related issues. We have highlighted specific actions that could be taken by different parties involved in research in order to help reduce the wider issues which affect publication bias.

## Supporting information

S1 File Survey given to academics/researchers. (PDF)

S2 File Survey given to editors. (PDF)

S1 Table Reasons given as to why each suggestion (Tables a-i) would be effective at reducing publication bias and the barriers or negatives to implementing this system.
(DOCX)

S2 Table Open ended answers for the question: “Overall, do you support the notion that the current system for publication should be changed to reduce publication bias–why or why not?”. (DOCX)

S3 Table Further suggestions offered by participants to reduce publication bias. (DOCX)
