# Supplementary material for: The perceived feasibility of methods to reduce publication bias
Source: PLoS One. 2017 Oct 24;12(10):e0186472. doi: 10.1371/journal.pone.0186472 (PMC5655535; doi:10.1371/journal.pone.0186472)
Supplement: S2 File — (PDF) [file pone.0186472.s002.pdf]

Publication bias_Editors

Page 1: Page 1

Thank you for taking the time to complete our survey, which should take between 4-15 minutes, depending on whether you wish to provide open ended answers which are all optional (required answers are marked "* *Required*"). **This survey is specifically for journal editors (including deputy, associate and assistant editors and those on editorial boards etc); if you are a student, peer-reviewer, academic or in another relevant academic or research position and wish to take part, please contact the primary researcher (details below) or complete the survey using the following URL:** https://sps.onlinesurveys.ac.uk/publication-bias_academics

You have been contacted as you are listed as a journal editor on your respective Journal's website, and we are interested in hearing the views of journal editors specifically. Your contact details were found on the Journal's website, or via an internet search using the details available on the Journal's website. Alternatively, you may have come across this survey via word-of-mouth, social media or other means.

We are interested in knowing different views regarding publication bias, and opinions on whether anything can or should be changed to tackle this problem. Specifically we would like to understand whether there are particular barriers to changing the current peer-review system in order to reduce publication bias. Many have expressed concerns regarding these biases but implementing change to address these issues has been limited. We therefore hope these results will highlight barriers to specific suggestions in the hope these issues they can be tackled.

We appreciate that you may not have a lot of time, but we feel the issue of

publication bias needs further exploration. By participating, you are potentially contributing to finding viable solutions to this problem. We do not foresee any risks by participating.

This project has been approved by the University of Bath REACH ethics committee (ref: EP 14/15 216). Please read all the instructions carefully and if you have any issues, please contact us using the details provided below. All data collected will be kept anonymous and confidential, and stored on a secure server in accordance with the Data Protection Act. Before continuing, you will be asked to provide the journal you are an editor for. Having this information allows us to analyse representativeness. Individuals will not be identifiable from this information; full anonymity is guaranteed. However, because this survey is anonymous, you will not be able to withdraw your answers once you have submitted them. If you have concerns regarding anonymity, please contact the primary researcher (details below).

By providing your journal name and continuing with this survey, you are giving your informed consent to participate, you are confirming you are a journal Editor, you understand you cannot withdraw your answers after submission, and you understand that your data will be used in accordance to data protection laws, and will not identify you as an individual.

Many thanks once again for participating.

Primary researcher: Harriet Carroll MSc (MRes/PhD student) (email: hac38@bath.ac.uk)

Supervisors: Dr Laura Johnson (email: laura.johnson@bristol.ac.uk)

Dr James Betts (email: j.betts@bath.ac.uk)

1. In order to give consent and continue to the survey, please provide your Journal name, then click 'Next' to continue ✱ *Required*

Page 2: Publication bias

Please read all questions carefully. Many questions are optional; only those marked "* *Required*" need to be answered in order to continue with the survey.

2. What factors do you think influence researchers to choose your journal for publication? Please rank in order of most influential (1) to least influential (5), providing at least the top 3 influencing factors in your opinion.

	Most appropriate content	Open access	Quality of journal (e.g. impact factor)	Turnaround time for publication	Other
1 (Most influential)	☐	☐	☐	☐	☐
2	☐	☐	☐	☐	☐
3	☐	☐	☐	☐	☐
4	☐	☐	☐	☐	☐
5 (Least influential)	☐	☐	☐	☐	☐

2.a. If 'other', please specify:

3. *Publication bias is when the published literature is systematically unrepresentative of the population of all completed research studies (Rothstein, Sutton & Borenstein, 2005). This can be due to several factors such as editors*

rejecting articles based on results, authors not submitting research based on results, or industry preventing or encouraging publication of research based on results, plus many other reasons. Considering this definition, have you heard of publication bias? ✱ *Required*

- Yes
- No

3.a. Do you think there is currently a problem of publication bias in the peer-reviewed literature? ✱ *Required*

- Yes
- No

4. Do you think peer-review in general is an effective means of publishing **quality** research? ✱ *Required*

- Yes
- No

4.a. Why or why not?

5. Do you think peer-review in general is an effective means of publishing **unbiased** research? ✱ *Required*

- Yes

○ No

5.a. Why or why not?

Page 3: Solutions

6. Below are 9 suggestions which aim to tackle publication bias. Please read each suggestion carefully, then using the Likert scale provided rate how effective you feel each suggestion would be in reducing publication bias (1 = not at all effective, through to 5 = extremely effective).

	* Required				
	1	2	3	4	5
MANDATORY PUBLICATION: As part of gaining ethical approval and/or by law, researchers would have to guarantee publication of their research, regardless of the findings.	☐	☐	☐	☐	☐
NEGATIVE RESULTS JOURNALS/ARTICLES: Having more journals specifically designed to accept research with negative, null and unfavourable results.	☐	☐	☐	☐	☐
OPEN REVIEWING: Requiring that journals name the reviewers and publish their comments with the final manuscript.	☐	☐	☐	☐	☐
PEER-REVIEW TRAINING AND ACCREDITATION: Requiring all peer-reviewers to attend peer-review training after which they would become accredited peer-reviewers on a peer-review database, which can also highlight potential conflicts of interest.	☐	☐	☐	☐	☐
POST-PUBLICATION REVIEW: Editors make a decision regarding the publication of an article. After publication, other researchers provide review comments which the authors can respond to. Although specific experts can be asked to conduct post-publication review, anyone is free to comment on all or part of the paper.	☐	☐	☐	☐	☐

PRE-STUDY PUBLICATION OF METHODOLOGY: Researchers publish full details of their planned methodology before commencing the research. The methods are then peer-reviewed to help ensure they are well justified. Once the study is completed, the full manuscript is peer-reviewed and published, regardless of the findings.	☐	☐	☐	☐	☐
PUBLISHED REJECTION LISTS: Journals would openly archive the abstracts of rejected manuscripts with a summary of why the paper was rejected.	☐	☐	☐	☐	☐
RESEARCH REGISTRATION: Researchers would be required to register their research on specific databases within a certain time frame of commencing the research. Registration would be compulsory for all research, and would include key aspects of the study design, including the primary and secondary outcomes and analysis plans.	☐	☐	☐	☐	☐
TWO-STAGE PEER-REVIEW: Authors initially submit only their introduction and methods to a journal. These get peer-reviewed, after which a decision is made regarding the study quality. If provisionally accepted, the authors would then submit the results and discussion for review. Rejection at this second stage would be justified by concerns over the quality of the reporting/interpreting of the results, but not according to the significance/direction of the results.	☐	☐	☐	☐	☐

6.a. From the list above, which suggestion do you think would be most effective at reducing publication bias? ✱ *Required*

- ○ Mandatory publication
- ○ Negative results articles/journals
- ○ Open reviewing
- ○ Peer-review training and accreditation

- Post-publication review
- Pre-trial publication of methodology
- Published rejection lists
- Research registration
- Two-stage peer-review

6.a.i. If you have time, please justify your answer

6.a.ii. What do you think the barriers would be to implementing this method?

6.a.ii.a. Do you think these barriers would be easy to overcome?

- Yes
- No

6.a.iii. Considering these barriers, would you support wide-spread implementation of this system?

- Yes
- No

6.a.iv. If there were no barriers to implementing this system, would you support wide-spread implementation?

- Yes
- No

6.a.v. How much do you agree or disagree with the following statements with regards to the method you have chosen as **most effective** at reducing publication bias (1 = Do not agree at all, through to 5 = Completely agree):

	✱ *Required*				
	1	2	3	4	5
This would take too much time for researchers/authors	☐	☐	☐	☐	☐
This would increase the amount of time it takes to review an article	☐	☐	☐	☐	☐
This would take too much time for editors	☐	☐	☐	☐	☐
To implement this would require too much change in the system (i.e. it would not be feasible)	☐	☐	☐	☐	☐
This would complicate the ethical approval process	☐	☐	☐	☐	☐
It would be hard to regulate such a system	☐	☐	☐	☐	☐
This is something I would consider for my journal	☐	☐	☐	☐	☐

6.a.vi. Any other comments or suggestions regarding this method of reducing publication bias?

6.a.vii. Do you have any comments regarding any of the other suggestions above? This can include your general thoughts, positives and negatives of the suggestion and/or potential barriers to implementing the suggestion. Please state clearly which suggestion(s) you are referring to.

Page 4: Final questions

7. Overall, do you support the notion that the current system for publication should be changed to reduce publication bias? ✱ *Required*

- Yes
- No

7.a. Why or why not?

8. Do you have any other suggestions which could reduce publication bias?

- Yes
- No

8.a. If ‘yes’, please outline your idea. If possible, also include any positives, negatives and barriers to implementing this method.

9. Generally speaking, in terms of changing the current publication process in order to reduce publication bias, are there any other barriers you can think of?

- Yes

○ No

9.a. If ‘yes’, please outline these barriers.

Page 5: Thank you

Thank you for taking the time to complete our survey - your responses are greatly appreciated. We hope the responses will help us understand the issues surrounding publication bias. If you have any queries regarding this project, please do not hesitate to contact us.

Please feel free to disseminate this survey to other editors who may be interested in participating. If you know of any other academics who may be interested, please contact us and we will provide you with a survey link specifically for non-editorial academics.

Harriet Carroll (email: hac38@bath.ac.uk)

Dr Laura Johnson (email: laura.johnson@bristol.ac.uk)

Dr James Betts (email: j.betts@bath.ac.uk)
