# Supplementary material for: The perceived feasibility of methods to reduce publication bias
Source: PLoS One. 2017 Oct 24;12(10):e0186472. doi: 10.1371/journal.pone.0186472 (PMC5655535; doi:10.1371/journal.pone.0186472)
Supplement: S1 Table — Reasons given as to why each suggestion (Tables a-i) would be effective at reducing publication bias and the barriers or negatives to implementing this system. (DOCX) [file pone.0186472.s003.docx]

**S1a-S1i Tables. Reasons given as to why each suggestion would be effective at reducing publication bias and the barriers or negatives to implementing this system.**

**S1a Table: Mandatory publication.**

| **Why most effective** | **Barriers/negatives** |
| --- | --- |
| Removes the choice of what can be published; everything gets published  Ed1, Ed7, Ed61, Ed68, Ed55  Ac49, Ac60, Ac93, Ac117, Ac146 | Time/workload  Ed1, Ed58, Ed61, Ed71, Ed55  Ac45, Ac49, Ac146, Ac158 |
| Encourages submission  Ed62 | Money  Ed1, Ed71  Ac63, Ac90, Ac146 |
| Least admin  Ac14 | Defining what needs to be published (e.g. a failed experiment vs one with undesirable results)  Ed1, Ed35  Ac47, Ac93, Ac135 |
| Covers many issues  Ac29, Ac69 | Journals won’t want [unfavourable] studies which are less likely to get cited (impact factor)  Ed7  Ac14, Ac106 |
| Reduces repetition of studies  Ac58 | Implementation and/or enforcement/regulation  Ed32, Ed61, Ed65, Ed72, Ed26, Ed49  Ac49, Ac50, Ac58, Ac90, Ac93, Ac122 |
| Changes perceptions on research quality  Ac63 | Researcher motivation  Ed58, Ed55  Ac49 |
|  | May not suit every discipline/type of research  Ed61, Ed9  Ac139 |
|  | Increased admin/bureaucracy  Ed61  Ac45, Ac69 |
|  | None  Ed 62 |
|  | Promoting it  Ed 65 Ed 71 |
|  | Removes quality filter  Ed5, Ed62, Ed49  Ac49, Ac54, Ac90, Ac117, Ac146 |
|  | Increases literature saturation  Ed5, Ed49  Ac12, Ac63 |
|  | May lead to reduced funding  Ed5 |
|  | Changing scientific culture  Ac29 |
|  | Doesn’t fully eliminate bias (e.g. authors and reviewers have opinions)  Ac49 |
|  | Industry/funder/researcher resistance / issues with patents  Ac29, Ac45, Ac60, Ac69, Ac90, Ac93, Ac135, Ac18 |
|  | Somewhat devalues results  Ac90 |

**S1b Table: Negative results articles/journals.**

| **Why most effective** | **Barriers/negatives** |
| --- | --- |
| Provides space for unfavourable findings  Ed10, Ed22, Ed24, Ed25, E35, Ed48, Ed57, Ed45  Ac2, Ac17, Ac38, Ac84, Ac105, Ac119 | Time  Ed10 |
| Reduces pressure for certain findings  Ed16  Ac92 | Money  Ed10, Ed24, Ed25, Ed35, Ed48, Ed10 |
| Null results are important  Ac7, Ac107 | Motivation of authors  Ed16, Ed35, Ed61  Ac32, Ac38, Ac95 |
| Reduces time wasting replicating research / informs future research  Ac13, Ac35, Ac92 | Impact factors / willingness of editors/publishers  Ed16, Ed22, Ed57, Ed64, Ed61, Ed10, Ed64  Ac2, Ac7, Ac52, Ac65, Ac66, Ac84, Ac92, Ac95, Ac105, Ac119, Ac158, Ac107 |
| Encourages publication  Ac32, Ac84 | Availability in science databases (will they include such journals?)  Ed24 |
| Highlights outliers in positive results  Ac35 | Increased reviewer burden  Ed25 |
| Gives authors credit for well conducted by negative research  Ac147 | More papers submitted/published / adds to literature saturation  Ed25, Ed10  Ac101, Ac122, Ac138 |
| Simple/not burdensome to implement  Ac107, Ac158 | Lack of audience  Ed49 |
|  | Changing perceptions of research (negative results)  Ac35, Ac107, Ac147 |
|  | Increased delays with publishing  Ac136 |
|  | None  Ac151 |

**S1c Table: Open reviewing.**

| **Why most effective** | **Barriers/negatives** |
| --- | --- |
| Doesn’t add bureaucracy or slow down the system  Ed41 | Less honest reviewers  Ed41 Ed26 |
| Improves fairness and objectivity of reviews  Ac37, Ac76, Ac83, Ac123, Ac132 | This does not address bias  Ed61, Ed5  Ac16, Ac90 |
| Avoid certain people always doing reviews  Ac128 | Reviewers would refuse / getting and maintaining reviewers  Ed10, Ed55  Ac4, Ac37, Ac83, Ac132, Ac140 |
| Increases accountability/transparency for reviews  Ac132, Ac140 | Author’s friends can review quickly and later posts won’t get as much attention  Ed35 |
|  | Nasty reviews / creates conflicts  Ed49  Ac122, Ac156, Ac91 |
|  | Editors  Ac155 |
|  | Changing dogma  Ac76, Ac123 |
|  | Lowers impact of peer-review  Ac122 |

**S1d Table: Peer-review training and accreditation.**

| **Why most effective** | **Barriers/negatives** |
| --- | --- |
| Increases value to the reviewer  Ed5 | Agreeing on standards  Ed5, Ed44, Ed45 |
| Increases value to the reviewed / increases review quality  Ed5, Ed45  Ac91, Ac125 | Implementing/managing a programme  Ed5, Ed8, Ed60 |
| Improves understanding of the role of peer-reviewer  Ed8 | Maintaining database  Ed8  Ac142, Ac73 |
| Helps identify conflicts of interest  Ed8  Ac125 | Time  Ed13, Ed63  Ac55, Ac142, Ac145, Ac115, Ac130 |
| Doesn’t change the system, just improves it  Ac142 | Resources  Ed45 |
|  | Effort  Ed45 |
|  | Money  Ed45  Ac125, Ac142, Ac145, Ac115 |
|  | May reduce pool of reviewers, cooperation of reviewers  Ed45, Ed63, Ed49 |
|  | No guarantee of reliable reviews  Ed60 |
|  | Enough training already  Ed26 |
|  | Designing and delivering training  Ac79, Ac125, Ac142 |
|  | No incentive / extra work for a voluntary role  Ac33, Ac72 |
|  | Reluctance  Ac33, Ac72 |
|  | Admin burden  Ac91 |

**S1e Table: Post-publication review.**

| **Why most effective** | **Barriers/negatives** |
| --- | --- |
|  | None  Ed29 |
|  | This would not reduce bias  Ed61 |
|  | Open to un-reviewed comments/abuse  Ed10, Ed55  Ac120 |
|  | Cost of moderation  Ed10 |
|  | Author reluctance to embrace it  Ed 5 |
|  | Still has reviewer biases  Ac120 |
|  | Has same issues as pre-publication peer-review  Ac120, Ac156 |
|  | Time  Ac59, Ac156 |
|  | No incentive  Ac59, Ac156 |

**S1f Table: Pre-study publication of methods.**

| **Why most effective** | **Barriers/negatives** |
| --- | --- |
| Judges research on design quality not outcomes  Ed37  Ac8, Ac43, Ac127 | Changing the system  Ed37 |
| Improves the quality of published literature  Ed56 | Post-study hypothesis generation  Ed40 |
| Useful to get feedback at this stage to improve methods pre-study  Ac22 | Loss of flexibility  Ed56 |
| Methods papers can stimulate new ideas for other researchers  Ac43 | Lack of interested audience, publishers, subscribers and authors to pay for it  Ed56, Ed26, Ed49 |
| Quicker publication  Ac43 | Increased workload for researchers (already done in grant application) and reviewers  Ed5  Ac8, Ac43, Ac48, Ac67 |
|  | Publishers would not approve  Ed37 |
|  | Adds to a complex system  Ed14 |
|  | Who would want to edit this journal?  Ed26 |
|  | Impact factors  Ed26 |
|  | Clutters literature  Ed73 |
|  | Time  Ac19 |
|  | Some might collect data first still  Ac19 |
|  | Others might take your idea  Ac67, Ac22 |

**S1g Table: Published rejection lists.**

| **Why most effective** | **Barriers/negatives** |
| --- | --- |
| Increases accountability from journals  Ed53 | Scale of the task, especially for big journals / extra work for editors and increased burden  Ed53  Ac39, Ac141 |
| Quick way to see what research has been done even if it wasn’t published; gets results out there  Ac39, Ac74 | Might shame authors  Ed49, Ed55  Ac122 |
| Easy to implement first step  Ac99 | Journal willingness and funds (doesn’t maximise profits)  Ac141, Ac74 |
|  | Lack of incentive  Ac74 |
|  | Moderating/ensuring fairness  Ac73 |
|  | None  Ac99 |
|  | Affecting chances of being published elsewhere  Ac73 |

**S1h Table: Research registration.**

| **Why most effective** | **Barriers/negatives** |
| --- | --- |
| Prevents data dredging  Ed15  Ac6, Ac42 | Resistance  Ed2, Ed15 |
| Attacks cause of problem  Ed15 | Time burden  Ed2, Ed20  Ac44, Ac98, Ac118 |
| Increases research accountability without clogging up literature  Ed26, Ed66  Ac44 | Can only be done with publicly funded studies  Ed3 |
| Low potential to be manipulated during research/publication  Ed59  Ac82 | Unsuitable for all studies/disciplines  Ed17, Ed61, Ed9  Ac122, Ac143 |
|  | Conflicts of interest and author bias  Ed20, Ed73 |
|  | Agreeing on acceptable structures/standardising/defining what needs to be published  Ed26, Ed54  Ac70 |
|  | Increased admin (especially for smaller studies)  Ed54 |
|  | Only works for pre-planned studies/analyses  Ed54, Ed61 |
|  | Money  Ed59  Ac98, Ac118 |
|  | Lack of incentive  Ed59 |
|  | Enforcement  Ed59, Ed66, Ed49  Ac159 |
|  | Getting funders/publishers on board  Ed59  Ac42 |
|  | Mitigates against efforts to try new things  Ed5 |
|  | Logistics  Ac82 |
|  | Increased admin / burden  Ac82 Ac70, Ac84 |
|  | May block patent opportunities  Ac50 |

**S1i Table: Two-stage review.**

| **Why most effective** | **Barriers/negatives** |
| --- | --- |
| Encourages submission of unfavourable findings  Ed9 | Harder to get reviewers  Ed9  Ac23, Ac53, Ac137 |
| Flexibility is maintained in analysis phase  Ac143 | Time (for reviewers/editors)  Ed19, Ed28, Ed33, Ed49  Ac18, Ac22, Ac36, Ac111, Ac112, Ac137 |
| Judges research on quality rather than results  Ac15, Ac18, Ac36, Ac51, Ac80, Ac89, Ac124, Ac139 | Higher rejection rates  Ed19 |
| Maintains peer-review  Ac16 | Increased work for reviewer  Ed49 |
| Easy to implement  Ac16 | Unsuitable for all disciplines  Ed61 |
| Reduced motives for submitting only favourable results  Ac143 | Bias at second stage of review  Ed57  Ac61, Ac64 |
| Reduces time for review  Ac30, Ac18 | Adds to a complex system  Ed14 |
| Long term and sustainable vs other solutions  Ac30 | Increased time to publication  Ac15, Ac20, Ac22, Ac31, Ac34, Ac53, Ac78, Ac111, Ac139, Ac73, Ac122 |
|  | Negative results not useful in every field  Ac117 |
|  | Cost  Ac124 |
|  | Journals/impact factors  Ac10, Ac64, Ac96, Ac110, Ac143, Ac150 |
|  | Might create a different type of bias (accepting ‘safe bets’ and not ‘long shots’)  Ac10 |
|  | None  Ac16, Ac26, Ac80 |
|  | Changing habits/attitudes / lack of will / too much effort  Ac30, Ac124, Ac153 |
|  | Time  Ac96, Ac100, Ac124, Ac143, Ac150, Ac153 |
|  | Increased admin  Ac89 |
|  | Logistics / difficult to implement  Ac150, Ac30 |
