# Supplementary material for: The perceived feasibility of methods to reduce publication bias
Source: PLoS One. 2017 Oct 24;12(10):e0186472. doi: 10.1371/journal.pone.0186472 (PMC5655535; doi:10.1371/journal.pone.0186472)
Supplement: S2 Table — (DOCX) [file pone.0186472.s004.docx]

**S2 Table. Open ended answers for the question: “Overall, do you support the notion that the current system for publication should be changed to reduce publication bias – why or why not?”.**

| **YES—why? (Editors n = 55/73; academics/researchers n = 142/160)** | **NO—why not? (Editors n = 8/73; academics/researchers n = 18/160)** |
| --- | --- |
| Publication bias exists and is problematic  Ed1, Ed17, Ed40, Ed54, Ed57, Ed61, Ed62, Ed73  Ac13, Ac15, Ac36, Ac39, Ac48, Ac49, Ac55, Ac69, Ac78, Ac83, Ac89, Ac114, Ac122, Ac127, Ac145, Ac147, Ac150 | Publication bias is not (that big of) an issue (field specific)  Ed9  Ac34, Ac59, Ac99, Ac103 |
| The publication process is flawed/there are problems, not in line with scientific principles (e.g. ethics, wasting resources)  Ed1, Ed37, Ed49, Ed54, Ed61  Ac12, Ac14, Ac29, Ac31, Ac37, Ac43, Ac48, Ac52, Ac63, Ac67, Ac74, Ac79, Ac89, Ac90, Ac108, Ac110, Ac122, Ac123 | Tweaked rather than changed; more consistently implemented  Ed10, Ed33, Ed38 |
| Should always be looking to improve the quality of literature  Ed45, Ed63, Ed67, Ed71, Ed72  Ac10, Ac29 | No evidence that high quality research is not being published  Ed13 |
| Lack of replication studies  Ac13, Ac41, Ac74 | Literature is already saturated with low quality/uninteresting studies  Ed13, Ed56 |
| Knowledge should benefit the public and not publications/headlines (public trust needs improving)  Ac23, Ac44, Ac79, Ac96 | No other solutions  Ed26  Ac120 |
| Industry has too much power  Ac108 | Too much effort to change for only a small number of bad papers  Ed35 |
|  | Bigger issues need addressing  Ed38, Ed44, Ed56  Ac93 |
|  | Change would result in an increase of poor quality papers  Ac146 |
|  | The current system is good/it works  Ac16, Ac137 |
|  | The current system changes/adapts  Ac99, Ac135 |
