# Supplementary material for: The perceived feasibility of methods to reduce publication bias
Source: PLoS One. 2017 Oct 24;12(10):e0186472. doi: 10.1371/journal.pone.0186472 (PMC5655535; doi:10.1371/journal.pone.0186472)
Supplement: S3 Table — (DOCX) [file pone.0186472.s005.docx]

**S3 Table. Further suggestions offered by participants to reduce publication bias.**

| **Suggestion (participant ID)** | **Comment/critique** |
| --- | --- |
| Editorial training (Ed5) | Echoes previous research [9] |
| Ethics boards to police research and ensure publication of data (Ac29) | Echoes previous research [9,10] |
| Excluding negative papers from impact factors (Ac111) | This assumes negative papers are not impactful and somewhat devalues their findings |
| Fully blinded reviewing (Ed22) | There are current issues with blinded reviewing (such as unfair reviews), though many argue this is a fairer system than open or single-blind reviewing, e.g. [7,41] |
| Investigators publishing reasons as to why they have not published their data (Ac47) | - |
| Linking registered research to submissions (Ed54) | Echoes previous research [9,10] |
| More credit for non-article contributors (e.g. data collectors who make their data available currently do not get the same credit as those who use the data and publish it in a paper) (Ac63) | This links with scientific culture and the way in which scientists are judged |
| Online searchable archives for low impact factor studies (Ed55) | This would leave either authors or reviewers to decide what papers are low impact and should be archived, thus may introduce its own bias |
| Open access repositories for data (Ed65) | - |
| Publication does not have to be in a journal (e.g. the increase in online publication options) (Ed62) | Removing a formal system may make it harder to distinguish what is genuine research. Some formality needs to remain (such as official research publishing websites) |
| Remunerating editors (Ed15) | Other research extends this sentiment to suggest full or part time editorial roles [9] |
| Remunerating reviewers (Ac39, Ac43) | This may lead to reviewers taking on too many reviews, or accepting articles they are not specialised in. Previous research has shown concern that payment may increase the cost of publication [41]. Incorporating review time into workloads may overcome this issue; alternatively others have proposed professional reviewers [9] |
| Rolling reviews/meta-analyses (to prevent several similar reviews with only a few added papers) (Ac10) | - |
| Using colleagues to replicate studies and verify findings upon publication (Ed22) | This would require extra time and funding which may not be available, though this may help for small inexpensive studies which are not as time consuming/expensive |
| Weighted impact factors to account for niche subjects with small readerships (Ac111) | This may still carry some issues that unweighted impact factors have (i.e. impact factors do not indicate the quality of the research) |
